# Diverse Roles and Targets of miRNA in the Pathogenesis of Testicular Germ Cell Tumour

**DOI:** 10.3390/cancers14051190

**Published:** 2022-02-25

**Authors:** Mrinal K. Das, Øyvind P. Haugen, Trine B. Haugen

**Affiliations:** 1 Department of Molecular Medicine, Institute of Basic Medical Sciences, University of Oslo, 0372 Oslo, Norway; 2 Institute of Oral Biology, University of Oslo, 0372 Oslo, Norway; oeyvind.haugen@gmail.com; 3 Department of Life Sciences and Health, OsloMet—Oslo Metropolitan University, 0130 Oslo, Norway

**Keywords:** testicular germ cell tumour, MicroRNA, gene regulation, function, male germ cell development

## Abstract

**Simple Summary:**

Testicular germ cell tumour (TGCT) is the most common malignancy among young males in many parts of the world. Although it is highly remediable, treatments lead to long-term comorbidities, and therefore, it warrants a better prognosis. The presence of several risk loci in non-coding regions supports a functional role of miRNAs in TGCT development, and recent studies point to the emerging roles of miRNA and the complexity of miRNA-mediated gene regulation in TGCTs. miRNAs may act as oncogenes or tumour suppressors in TGCT by regulating targets involved in cell proliferation, apoptosis, and metastasis. Here, we summarise the gene regulation and function of miRNAs involved in TGCT pathogenesis.

**Abstract:**

Testicular germ cell tumour (TGCT) is the most common cancer type among young adults in many parts of the world. Although the pathogenesis of TGCT is not well understood, the involvement of heritable components is evident, and the risk is polygenic. Genome-wide association studies have so far found 78 susceptibility loci for TGCT, and many of the loci are in non-coding regions indicating the involvement of non-coding RNAs in TGCT pathogenesis. MicroRNAs (miRNAs), a class of non-coding RNAs, have emerged as important gene regulators at the post-transcriptional level. They are crucial in controlling many cellular processes, such as proliferation, differentiation, and apoptosis, and an aberrant miRNA expression may contribute to the pathogenesis of several cancers, including TGCT. In support of this notion, several studies reported differential expression of miRNAs in TGCTs. We previously demonstrated that miRNAs were the most common group of small non-coding RNAs in TGCTs, and several functional studies of miRNAs in TGCTs suggest that they may act as either oncogene or tumour suppressors. Moreover, individual miRNA targets and downstream pathways in the context of TGCT development have been explored. In this review, we will focus on the diverse roles and targets of miRNAs in TGCT pathogenesis.

## 1. Introduction

Despite the rare incidence, testicular germ cell tumour (TGCT) is the most common cancer type among young men in many parts of the world [1]. TGCT is highly remediable with approximately a 95% survival rate, mainly because of the unique responsiveness of TGCT to platinum-based chemotherapy [2]. However, chemotherapeutic treatment is associated with long-term morbidities such as infertility, metabolic syndrome, and cardiovascular diseases [3]. It is believed that the foundation of TGCT happens with the developmental arrest of foetal gonocytes, followed by the development of the precursor cells called germ cell neoplasia in situ (GCNIS) [4]. Though the pathogenesis of TGCT is not well understood, disruptions in the early development of the testis are evident. Due to the pluripotent nature and the histological heterogeneity of TGCTs, the underlying molecular mechanisms appear to be multifaceted.

TGCT development is strongly influenced by inherited genetic factors since sons of TGCT patients show a four- to sixfold increased risk, and brothers of TGCT patients have an eight- to tenfold increased risk of developing the tumours [5,6]. According to genomic datasets, over three-quarters of the inherited genetic risk may be transmitted through common variation [6]. The inherited TGCT risk is largely polygenic [7], and genome-wide association studies have revealed 78 susceptibility loci for TGCT [8]. Functional studies show that a large part of the risk genes is related to male germ cell development, sex determination, and genomic integrity [9]. Many of the loci are in non-coding regions, indicating the potential involvement of non-coding RNAs in TGCT pathogenesis [10,11,12].

MicroRNAs (miRNAs), a class of non-coding RNAs, are involved in multiple cellular processes, and controlled expression of miRNAs is essential for normal cell development, cell differentiation, and homeostasis [13]. Aberrant miRNA expression can lead to diseases, including cancer. So far, several studies have reported the differential expression of miRNAs in TGCTs, although subsequent functional studies of those miRNAs in TGCTs are few. This is partly due to the lack of suitable model systems for TGCT since there are differences in types and occurrence of testicular cancer between animals and humans [14,15]. Even so, results from animal and in vitro studies may contribute to the understanding of the mechanisms behind TGCT development. In this review, we give a synopsis of functional miRNAs and their targets involved in TGCT pathogenesis. 

## 2. Male Germ Cell Development

In human males, germ cell development starts around 4−5 weeks of gestation, when primordial germ cells (PGCs) migrate to the hind gut epithelium under control of the SCF/c-KIT signalling system [16]. PGCs colonize the genital ridges, which form the gonads, and become gonocytes. At the 10th week of gestation, gonocytes turn into intermediate cells upon migration from the centre to the periphery of the seminiferous tubules. After week 15, the intermediate cells differentiate into spermatogonia and from the 18th week, the spermatogonia constitute the main part of the germ cell population [17]. In humans, spermatogonia show at least two subtypes: undifferentiated (type A) and differentiated (type B). Type A spermatogonia differentiate and form type B spermatogonia, which undergo mitotic proliferation until puberty [18,19]. The meiotic process is initiated by the formation of spermatocytes. During the second meiotic division, haploid round spermatids are formed, turning into elongated spermatids during spermiogenesis [20]. Human spermatogenesis takes 74 days and produces 16 spermatids from each spermatogonial cell [21]. The sperm is released into the lumen of the seminiferous tubules and transferred to the epididymis for final maturation [22].

Considering molecular features, the derivatives of germ cell populations express different markers of pluripotency with different epigenetic modifications. Gonocytes express OCT3/4, NANOG, c-Kit, and placental alkaline phosphatase (PLAP) while intermediate cells express a proliferation marker, called proliferating cell nuclear antigen (PCNA). Around one year of post-natal life, spermatogonia no longer express foetal markers, but germ-cell-specific markers such as MAGE4A, VASA, and testis-specific protein Y-encoded gene (TSPY) [23,24,25,26]. Genes associated with germ cell development are tightly regulated by epigenetic changes and miRNAs [27,28]. miRNAs are found to be crucial for spermatogenesis and may play an important role during all stages of spermatogenesis by regulating the expression of the target genes [29,30,31,32]. Several studies also demonstrated that removal of Dicer, a key component of miRNA biogenesis, resulted in disrupted spermatogenesis [32,33,34]. Furthermore, one of these studies showed that removal of DICER1 at the early onset of male germ cell development caused infertility, due to various accumulative defects in the meiotic and post-meiotic stages [33]. Since miRNAs play a critical role in spermatogenesis, disturbances in miRNA function may lead to TGCT development.

## 3. TGCT Development

**Figure 1:**
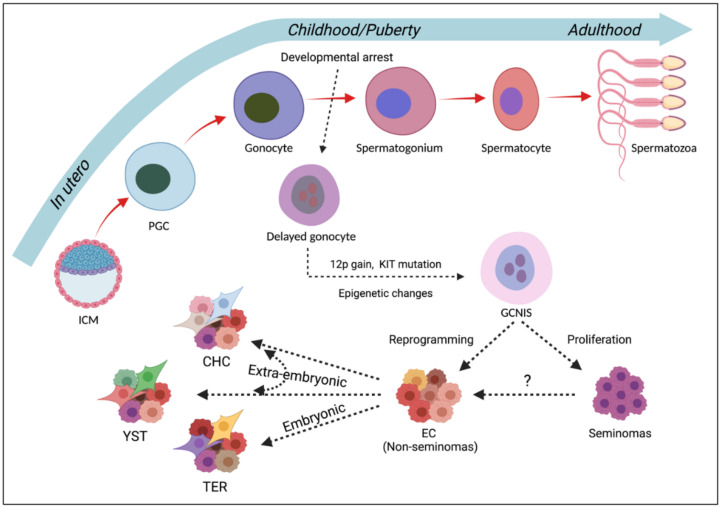
Germ cell development vs. TGCT development. ICM—inner cell mass, PGC—primordial germ cell, GCNIS—germ cell neoplasia in situ, EC—embryonal carcinoma, CHC—choriocarcinoma, YST—yolk sac tumour, TER—teratoma.

TGCT development displays a common pathologic precursor, the germ cell neoplasia in situ (GCNIS)—previously known as carcinoma in situ, testicular intraepithelial neoplasia, or intratubular germ cell neoplasia unclassified [26]. GCNIS is believed to be an embryonic germ cell, referring to a primordial germ cell or a gonocyte that fails to differentiate into a spermatogonium. These gonocytes accumulate oncogenic adaptations through childhood and puberty and develop into GCNIS and subsequently invasive TGCT in young adults (Figure 1).

Despite expressing pluripotency markers, GCNIS cells remain undifferentiated until puberty. During this period, chromosomal aberrations may occur in GCNIS cells that affect genes related to proliferation and differentiation. Growth signals and hormones induce the proliferation of GCNIS in puberty, which may result in malignancy [35]. The main histological subtypes of TGCTs are seminoma and non-seminoma. Seminomas resemble undifferentiated gonocytes and constitute ~55% of TGCT cases, peaking at ages 35−39 years. Non-seminomas make up ~45% of TGCTs, are generally more aggressive, and have a younger age of diagnosis (25−29 years). Non-seminomas are heterogeneous in composition, reflecting their differentiation into embryonal carcinoma (EC), teratoma, choriocarcinoma, and yolk sac tumour [36]. Studies on chromosomal aberrations in invasive seminoma and non-seminoma neoplasms demonstrated that 80–100% of these tumours and GCNIS cells adjacent to cancer exhibited a gain of the short arm of chromosome 12 [37], usually in the form of an isochromosome, i(12p) chromosome [38]. The gain of 12p is regarded as a key factor for TGCTs to acquire invasiveness. On the contrary, GCNIS cells, which are relatively distant from the cancerous zone, normally do not present short arm of chromosome 12 gain. 

The underlying mechanism of invasive TGCT development from premalignant GCNIS is yet to be resolved. Whether GCNIS develops into seminoma and non-seminoma separately or if non-seminoma derives from seminoma, is still debatable. According to the first proposition, phenotypically heterogeneous GCNIS consists of cells at different stages of progression [39,40], and KIT mutations are observed in a subset of seminoma, but not in EC [41]. The second proposition is mainly based on the phenotypic resemblance of seminoma to GCNIS, occurrence of aneuploidy [42], cytogenetics [43], and pathomorphology [44]. 

## 4. miRNA Target Regulation and Function

**Figure 2:**
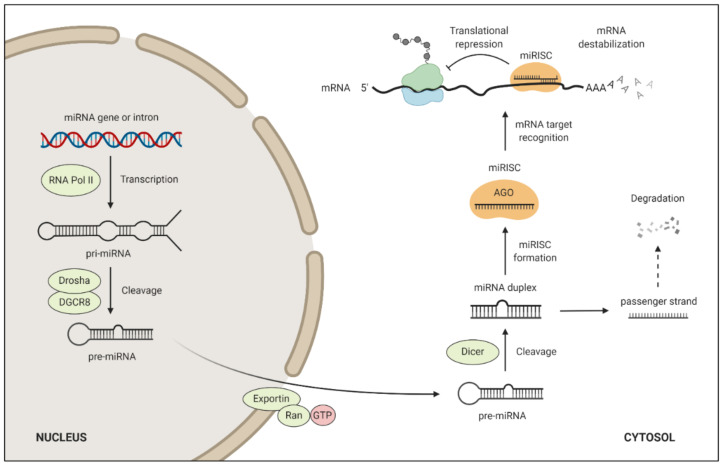
miRNA biogenesis and target regulation. miRNA biogenesis pathway begins with the transcription of a much larger primary miRNA transcript (pri-miRNA). The pri-miRNA is processed into a smaller pre-miRNA by the endonuclease Drosha and subsequently transported out of the nucleus through Exportin-5. In the cytosol, the pre-miRNA encounters the endonuclease Dicer, which cleaves off the hairpin loop, leaving a pre-miRNA duplex. One of the strands, constituting the mature ~22 bp miRNA, then associates with an Argonaut protein to form the miRNA-induced silencing complex (miRISC). Once bound to the targeted mRNA, miRISC induces translational repression and mRNA destabilization, thereby downregulating gene expression.

miRNAs are small endogenous RNA molecules of ~22 nucleotides in length, too small to code for any protein. Instead, they regulate protein-coding genes on the post-transcriptional level through repression of targeted mRNAs [45]. The biogenesis and target regulation of miRNA are shown in Figure 2. It is estimated that ~60% of the human protein-coding genes are conserved targets of miRNAs [46], which implies that miRNAs are important for normal development [47,48]. Deletions of the fundamental miRNA biogenesis factors Dicer [49] and Drosha [50] have been found to be lethal in mouse embryos. The miRNA functions as a guide RNA for the Argonaut protein to repress specific mRNAs. Although examples of miRNAs binding to mRNA through extensive base-pairing exist, leading to slicing of the mRNA, this regulatory mechanism is rather rare in animals [51]. Instead, most animal miRNAs bind to the 3′-UTR of mRNAs through a limited portion of their sequence, known as the ‘seed’, involving nucleotides 2–7 counted from the miRNA 5′-end. Base-pairing between the miRNA seed and the complementary target site causes translational repression and accelerated mRNA destabilization [45]. Whereas translational repression occurs more rapidly, most of the steady-state repression is due to accelerated mRNA destabilization [52].

One miRNA can silence hundreds of genes due to the small size of the seed required for target interaction [53], and hence, entire cellular pathways can be regulated by individual miRNAs [54] or miRNA clusters [55]. Multiple miRNAs can also regulate the same gene [54], and miRNA binding to neighbouring sites on a target mRNA can result in cooperative repression [13]. Moreover, the strength of repression can differ between target sites and is dependent on several features concerning the miRNA and mRNA [56]. This means that very complex mRNA expression profiles can be seen in a cell, depending on which miRNAs are being present and the degree to which they are expressed. Changes in miRNA expression can trigger a developmental transition but can also lead to disease. Abnormal miRNA expression is frequently found in cancer, and miRNAs may function as oncogenes or tumour suppressors depending on which protein-coding genes they regulate and the cellular context [57]. 

## 5. miRNAs in TGCTs

**Figure 3:**
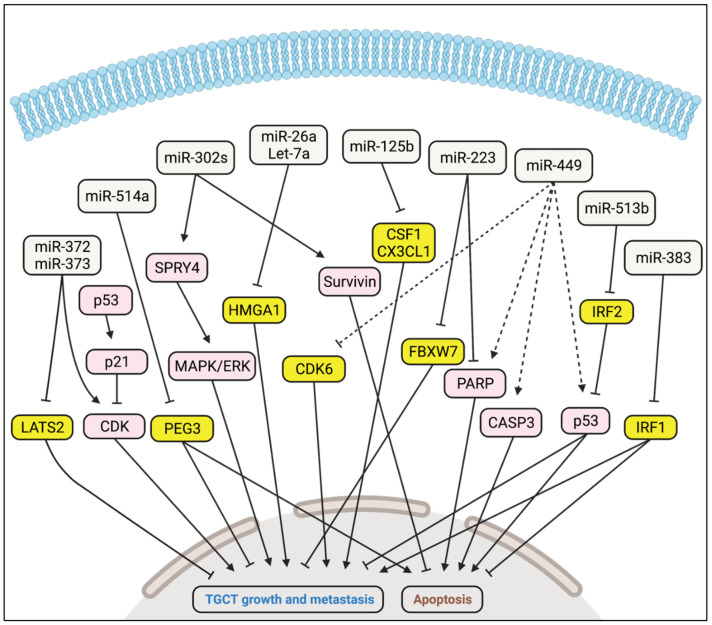
miRNA signalling pathways related to TGCT pathogenesis. Genes affected by miRNAs: yellow boxes represent direct targets, and the pink boxes represent indirect targets involved in TGCT pathogenesis. Solid lines indicate activation or inhibition based on evidence, whereas broken lines indicate hypothesized mechanisms.

Altered expression of miRNAs in TGCTs indicates that miRNAs may play a key role in TGCT pathogenesis [58,59,60,61]. Our group showed, for the first time, that miRNAs were the most common group of sncRNAs in TGCTs [61]. In the same study, we also demonstrated that miRNA expression profiles differed between malignant and normal testis tissue. However, few studies of the function of miRNAs in TGCTs have been conducted. In the following sections, we describe the roles of miRNAs and their targets in TGCT development that have been reported so far. Table 1 summarises miRNA functions and targets involved in TGCT development. A representation of signalling pathways related to TGCT development is shown in Figure 3.

### 5.1. Oncogenic Activity

Several studies have reported high expression of the miR-302/367 and miR-371-373 clusters in tumour tissues and serum samples from TGCT patients [58,59,60,61]. The findings indicate an oncogenic role for these miRNAs in TGCT development. Voorhoeve et al. demonstrated that miR-372 and miR-373 acted as TGCT oncogenes through inhibition of a tumour suppressor, LATS2 [62]. They found that TGCTs expressing miR-372 and miR-373 only contained wildtype p53, while mutated p53 alleles were present in a subset of TGCTs and cell lines which were miR-372 and miR-373 negative. Based on these findings, they hypothesized that mutation in p53 during TGCT development has no selective advantage when the miR-371-373 cluster is expressed. The role of the miR-302/367 cluster in TGCTs was unclear until our group reported that miR-302a, miR-302b, and miR-302c may act as oncogenes in these tumours. We found that miR-302/367 and miR-371-373 clusters were highly expressed in TGCTs [61,63]. In particular, the expression of miR-302/367 cluster members were highest in the embryonal carcinoma subtype [63].We also studied the role of miR-302/367 cluster in two TGCT cell lines containing mainly embryonic carcinoma subtype (833K and NT2-D1) and found a decline in proliferation of NT2-D1 cells when inhibiting the expression of the three members of miR-302 cluster; miR-302a-3p, miR-302b-3p, and miR-302c-3p. Treatment of the 833K and NT2-D1 cells with the cytotoxic drug cisplatin also reduced the expression of these three miRNAs. Furthermore, inhibition of miR-302s resulted in the decreased expression of SPRY4, a regulator of MAPK/ERK and PI3K/Akt signalling pathways. In another study, we found SPRY4 to be highly expressed in TGCTs while absent in normal testis [64]. Knockout of SPRY4 in TGCT cells reduced cell growth, migration, invasion, and phosphorylation of ERK1/2 and Akt. Inhibition of miR-302b-3p and miR-302c-3p also reduced the phosphorylation of ERK1/2 in 833K and NT2-D cells [63]. The finding that suppression of miR-302s inhibited the activation of MAPK/ERK signalling pathway had as far as we know, not been reported in any cancer type. Our results with TGCT cells suggest that miR-302s may act as oncogene in TGCTs by targeting a tumour suppressor gene upstream SPRY4. We also demonstrated that the expression of survivin, an inhibitor of apoptosis, was suppressed by inhibition of miR-302s in TGCT cells [63]. High expression of survivin is a hallmark of practically all human tumours including TGCTs [65,66]. Another miRNA, miR-223-3p, has also been found to be expressed higher in TGCT compared with normal testes, and its upregulation may promote cell proliferation and inhibit apoptosis [67]. Shimizu et al. reported that miR-223-3p triggered its oncogenic role in TGCT through repression of FBXW7, a substrate-recognition component of the tumour suppressor SCF–ubiquitin–ligase complex [68]. In line with this, Liu et al. showed a negative correlation between miR-223-3p and FBXW7 mRNA expression in TGCT [67]. 

### 5.2. Tumour Suppressor Activity

Lize et al. showed that miR-449 was highly expressed in normal testes, while robustly downregulated in TGCTs, indicating a putative tumour suppressor role [69]. They found that E2F1-inducible miR-449a suppressed cell proliferation and promoted apoptosis in HCT116 cells, a human colon cancer cell line. The transcription factor E2F1 plays a key role in cell proliferation, and its uncontrolled activity leads to malignant growth. They also showed that miR-449a reduced the expression of cell cycle protein CDK6, thus possibly counteracting the oncogenic activity of E2F1. De Martino et al. suggested a role of Let-7a and miR-26a in seminomas [70]. They referred to the Cancer Genome Atlas database, showing low expression of Let-7a and miR-26a in human seminoma, and an inverse corelation with HMGA1 expression. They also found that Let-7a- and miR-26a-mediated repression of HMGA1 resulted in reduced cell growth in the seminoma cell line TCam-2. In another study, miR-125b levels were found to be downregulated in TGCT [71]. Overexpression of miR-125b inhibited the TGCT growth by targeting CSF1 and CX3CL1, which are known tumour-derived chemokines, involved in the recruitment of macrophages to the neoplastic sites. Another miRNA, miR-514a, was found to be downregulated in TGCTs [72]. Overexpression of miR-514a in TGCT cells resulted in reduced cell viability and increased apoptosis through targeting the anti-apoptotic paternally expressed gene 3 (PEG3), an activator of NF-κB pathway. miR-383 was also found to be downregulated in TGCT cells, and overexpression of miR-383 resulted in decreased proliferation and increased apoptosis via targeting IRF1, a pro-mitogenic factor [73]. In a follow-up study, Lü et al. found that the transcription of miR-383, as well as miR-320a, was repressed by the co-operative action of long intergenic non-coding RNA162 and nucleolin, thereby increasing proliferation and inhibiting apoptosis [74]. Wang et al. described miR-513 as a tumour suppressor in testicular embryonal carcinoma cells where miR-513 triggered p53 expression by targeting IRF2 [75]. 

There are many other miRNAs that are found to be differentially expressed in TGCTs, but their functions are yet to be explored. Particularly, three miRNAs from the chromosome 19 miRNA cluster (miR-517a, miR-519a, and miR-519c) showed lower expression in seminomas and higher expression in non-seminomas than in normal testis [76]. Other miRNAs, such as miR-200, miR-141, miR-367, miR-512-3p, miR-515, miR-518, and miR-525, were found to be up-regulated, while miR-99a, miR-100, and miR-145, have been found to be down-regulated in TGCTs [61,77,78].

**Table 1:** Targets and functions of miRNAs involved in TGCT pathogenesis.

miRNA	Target	Function in TGCT	Reference
miR-372 miR-373	LATS2	Promote TGCT growth and metastasis	[62]
miR-302a miR-302b miR-302c	Unknown	Promote TGCT growth and metastasis via SPRY4 and MAPK/ERK	[63]
miR-223	FBXW7	Promotes TGCT growth	[67]
miR-449	CDK6	Inhibits TGCT growth	[69]
miR-26a Let-7a	HMGA1	Inhibit TGCT growth	[70]
miR-125b	CSF1, CX3CL1	Inhibits TGCT growth	[71]
miR-514a	PEG3	Inhibits TGCT growth	[72]
miR-383	IRF1	Inhibits TGCT growth	[73]
miR-513b	IRF2	Inhibits TGCT growth	[75]

## 6. Conclusions

The incidence of TGCT has increased several folds in many countries over the last 60 years for unknown reasons, and the molecular mechanisms underlying TGCT development are poorly understood. The presence of several risk loci in non-coding regions supports a functional role of miRNAs in TGCT development, and recent studies point to the emerging roles of miRNA and the complexity of miRNA-mediated gene regulation in TGCTs. As described in this review, miRNAs may act as oncogenes or tumour suppressors in TGCT by regulating targets involved in cell proliferation, apoptosis, and metastasis. However, the mode of action of miRNAs as regulators in TGCT development are still evolving. A role of miRNA as a ligand has been discovered in immune cells where tumour-secreted miRNAs directly bound to Toll-like receptors, which mediated a pro-metastatic inflammatory response [79]. Whether miRNAs may also act as ligands to activate the signalling pathways in TGCT remains to be elucidated.

It is a challenge to study the function of miRNAs in TGCT development due to the lack of appropriate animal models and the limitations of the TGCT cell lines and xenograft models used. TGCT patient-derived induced pluripotent stem cells may bring up a new possibility of disease modelling. Techniques such as the CRISPR/Cas9 system to tailor genomic modifications in iPSC lines also offer new opportunities for understanding the early onset of TGCT and tumour progression.

## Abbreviation

EC	Embryonal carcinoma
GCNIS	Germ cell neoplasia in situ
miRNA	MicroRNA
miRISC	miRNA-induced silencing complex
PGC	Primordial germ cell
pri-miRNA	Primary miRNA transcript
TGCT	Testicular germ cell tumour
